# Fermented Kiwifruit By-Product as Experimental Biostimulant for Soilless Mini-Plum Tomato Cultivation

**DOI:** 10.3390/plants15010082

**Published:** 2025-12-26

**Authors:** Anna Agosti, Alessia Levante, Jasmine Hadj Saadoun, Samreen Nazeer, Lorenzo Del Vecchio, Leandra Leto, Massimiliano Rinaldi, Rohini Dhenge, Martina Cirlini, Camilla Lazzi, Benedetta Chiancone

**Affiliations:** 1 Department of Food and Drug, University of Parma, Viale Parco Area delle Scienze 27/A, 43124 Parma, Italy; alessia.levante@unipr.it (A.L.); jasmine.hadjsaadoun@unipr.it (J.H.S.); samreen.nazeer@unipr.it (S.N.); lorenzo.delvecchio@unipr.it (L.D.V.); leandra.leto@unipr.it (L.L.); massimiliano.rinaldi@unipr.it (M.R.); rohinivijay.dhenge@unipr.it (R.D.); martina.cirlini@unipr.it (M.C.); camilla.lazzi@unipr.it (C.L.); 2 Institute of Biophysics, National Research Council (CNR), Via Ugo La Malfa 153, 90146 Palermo, Italy

**Keywords:** agri-food waste, bacterial community, biostimulant, fermented kiwifruit, fruit quality, plant growth, soilless cultivation, tomato

## Abstract

Biostimulants boost plant growth, productivity, and nutrient retention, and can be produced from agri-food waste via microbial fermentation. In this study, undersized and unsold kiwifruits were fermented with *Lactiplantibacillus plantarum* to produce a fermented kiwifruit-based biostimulant (FKB). FKB was applied to soilless tomato plants (cv. Solarino) at two concentrations (50 and 100 mL L^−1^) at the root level, every two weeks throughout the crop cycle. Fruits were analyzed for technological and chemical parameters, including color, texture, total soluble solids, titratable acidity, sugar/acid ratio, pH, electrical conductivity, total polyphenol content, antioxidant activity, and lycopene concentration. Additionally, metataxonomic analysis characterized the substrate microbial community at the beginning and the end of cultivation. Overall, the results indicate a dose-dependent effect of FKB on fruit quality parameters, with the highest concentration showing the most pronounced effects, specifically for the fruit firmness (8.02 N for FKB at 100 mL L^−1^ vs. 7.25 N for the Control). Moreover, both tested concentrations were associated with increased antioxidant activity (on average +28%), and lycopene content (on average +57%) compared with the Control fruits. While overall microbial diversity remained largely unchanged, the relative abundance of bacterial taxa associated with nutrient cycling and plant–microbe interactions was modulated by the biostimulant, indicating subtle but potentially functionally relevant shifts in the rhizosphere microbiota. These findings suggest that fermented kiwifruit biomass can serve as an effective biostimulant, improving both fruit quality and the functional structure of the rhizosphere microbial community in soilless tomato cultivation.

## 1. Introduction

Tomato (*Solanum lycopersicum* L.) is a cornerstone crop of Mediterranean horticulture and a key economic driver in Italy, particularly in Emilia-Romagna, which hosts most of the national production and makes Italy the leading tomato producer in Europe [1,2]. Given the economic relevance of tomato cultivation and the increasing pressure to improve the sustainability of production systems, the adoption of innovative and environmentally friendly agronomic practices, such as identifying effective alternatives, particularly of organic origin, for synthetic fertilizers, has become a priority. In light of these considerations, a growing interest has emerged in both the commercial and scientific sectors toward plant biostimulants [3,4], whose modes of action are associated with the activity of bioactive metabolites that stimulate primary and secondary plant metabolism, improving productivity and fruit’s organoleptical qualities [5,6], as well as nutrient trapping in the soil and rhizosphere [7].

According to Du Jardin et al. [8], there are different types of organic biostimulants on the market, namely protein hydrolysates, polysaccharides, algal extracts, fulvic and humic acids, plant extracts, and microorganisms. Biostimulants can be obtained from a plethora of starting materials, deriving from aquatic and terrestrial ecosystems, including microorganism waste and agri-food waste.

The growing generation of agri-food waste, particularly from fruits and vegetables, which show the highest discard rates at both the retail and consumer levels [9,10], represents a pressing environmental and economic challenge. Using agri-food waste as a matrix for biostimulant production is therefore a win-win strategy: it not only addresses the issue of disposing of the large amount of organic biomass generated along the food supply chain [11] but also provides an additional source of income for agri-food companies and promotes the integration of agriculture within a circular economy framework.

According to an FAO report (2025) [12], global kiwifruit production is estimated at 4.43 million tons per year, with Europe accounting for about 18% of total production and Italy representing approximately 9% of global production. Due to the strict quality standards, the kiwifruit supply chain globally generates a total of 1 million tons of waste per year, from harvest to retail [13]. Notably, 13% of this waste occurs at the retail level [14], largely due to the high standards and preferences of consumers when selecting products. Kiwifruit waste is a material with a lot of potential, so much so that it has been used to generate bioelectricity in microbial fuel cell technology [15] and, due to its wealth of compounds of interest, as a matrix to produce extracts to be used in several productive sectors [16].

As with other types of waste and by-products, one strategy for kiwifruit by-product valorization is the fermentation process. Microorganisms can use these materials as nutrients, which leads not only to profound changes in nutritional and aromatic profiles but also to the production of various metabolites with antimicrobial or functional properties. Fermentation-based valorization of fruit and vegetable by-products is increasingly recognized as a key component of the circular bioeconomy, providing an eco-efficient route for converting agro-industrial residues into bioactive compounds [17]. In recent years, there has been a growing interest in the use of agri-food residues as fermentable raw materials by exploiting the ability of some microorganisms, such as lactic acid bacteria (LAB), to modulate their bioactive compound profile [18,19,20]. For example, LAB have been successfully used to ferment agri-food by-products, such as okara, enhancing their content of bioactive compounds and enzymatic activities, and producing functional materials suitable as soil biostimulants [21]. Recent studies have further demonstrated the potential of fermented kiwifruit waste as an effective biostimulant source. Nazeer et al. [22] reported that fermented kiwifruit by-products significantly improved the morpho-physiological and productive performance of *Fragaria* spp. grown hydroponically, while Galaverni et al. [23] showed that fermented kiwifruit juice, obtained through LAB fermentation, enhanced tomato quality and stress resilience under water stress, in open-field conditions.

This study aimed to investigate the potential use of fermented kiwifruit biomass, obtained from undersized fruits discarded due to their small size, as a biostimulant in soilless tomato cultivation. The performance of this biostimulant was evaluated not only by monitoring plant vegetative parameters and assessing fruit quality, but also by analyzing its effects on the soil microbial community. This dual approach provides a comprehensive understanding of how fermented agri-food waste can influence both plant growth and the structure and function of rhizosphere microbial populations, offering insights into the mechanisms through which biostimulants derived from agrifood waste may enhance sustainable crop production.

## 2. Results

This study aimed to evaluate the effects of a biostimulant derived from fermented kiwifruit biomass (FKB), applied at two concentrations (50 mL/L—50FKB—and 100 mL/L—100FKB), on the vegetative growth, yield, and quality traits of Solarino, an indeterminate tomato cultivar grown under soilless conditions.

### 2.1. Plant Height Development and Growth Dynamics in Response to Fermented Kiwifruit Biomass Treatments

Considering plant height, statistically significant differences were detected at weeks 2, 3, and 4 during flowering and at the beginning of fruit setting, respectively. Specifically, it was noted that in weeks 2 and 3, the 100FKB treatment resulted in a statistically higher height (respectively, 35.3 ± 0.9 cm and 53.0 ± 1.0 cm) compared to the non-treated plants (Control) (respectively, 32.0 ± 0.6 cm and 49.6 ± 1.3 cm). At week 4, the height of plants treated with 100FKB (99.8 ± 0.7 cm) was comparable to the Control (103.3 ± 2.6 cm), while the height of plants treated with 50FKB was characterized by a statistically lower height (92.6 ± 2.6 cm). In the following weeks, until the topping, the plants had a comparable height, reaching on average 226.70 ± 4.71 cm (Table S1).

**Figure 1:**
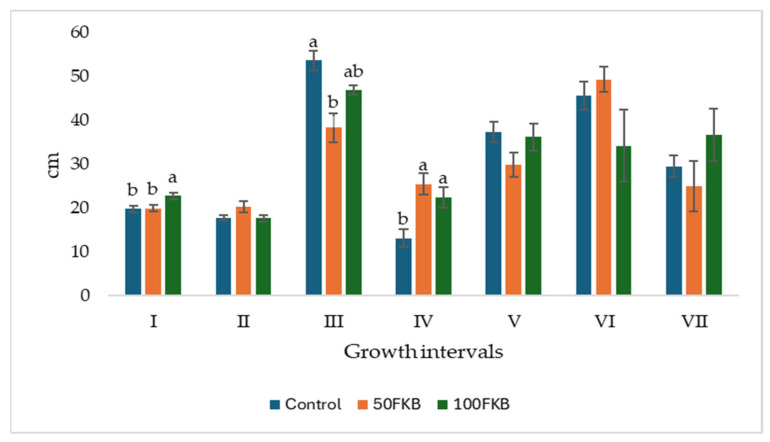
Trends in tomato plant height in response to various treatments, during 8 weeks of monitoring. Control: 0 mL L^−1^ fermented kiwifruit biomass (FKB); 50FKB: 50 mL L^−1^ FKB; 100FKB: 100 mL L^−1^ FKB. Interval I: formation of side shoots, Interval II and III: Inflorescence emergence, Interval IV: Flowering, Interval V: Development of fruits, Interval VII and VIII: Ripening of fruit and seeds. Data are expressed as mean ± standard error. Within each interval, bars marked with different letters are significantly different according to Tukey’s test (*p* < 0.05). Intervals without letters indicate an absence of statistically significant differences among treatments.

Considering the weekly plant height increments, the trend in growth dynamics revealed distinct responses among treatments throughout the growing period, until plant topping (Figure 1). In the first interval (Interval I), corresponding to side shoot formation, plants treated with 100FKB showed a significantly higher height increment than the Control and 50FKB, suggesting an early stimulatory effect of the higher concentration. No significant differences were observed in Interval II. The greatest increase in plant height occurred in Interval III, coinciding with inflorescence emergence, when Control plants exhibited significantly greater elongation compared to treated 50FKB. In contrast, during Interval IV, corresponding to the flowering stage, both biostimulant treatments (50FKB and 100FKB) determined significantly higher height increments compared to the Control, indicating a positive vegetative response during this phase. In the final intervals (VI and VII), associated with fruit growth and ripening, the rate of height increase was not influenced by the treatments; thus, no statistically significant differences were observed.

Weekly stem-diameter measurements indicated a consistent and expected growth trend. Additionally, the stem diameter, recorded weekly until week 8, showed a similar growth trend for all plants, both treated and untreated with FKB, reaching at week 8 (the beginning of harvesting), values of 1.76 ± 0.10 cm for Control plants and 1.64 ± 0.29 cm for treated plants. The number of leaves was recorded weekly until the beginning of defoliation, and statistical analysis did not show any differences among the treatments (Table S2).

### 2.2. Effects of Fermented Kiwifruit Biomass on Yield and Fruit Morphology

Regarding yield, no statistically significant differences were observed (*p* = 0.076) among the treatments in either the average number of fruits harvested per plant at each harvest date (*p* = 0.151) or the total weight of fruits harvested per plant (*p* = 0.456), as reported in Table 1.

The analysis of the data on fruit morphological parameters, both in terms of Longitudinal Diameter (LD) and Equatorial Diameter (ED), revealed that fruits from treated plants did not differ significantly from those harvested from Control plants, with *p*-values of 0.561 and 0.633, respectively (Table 1). Furthermore, the fruit fresh weight (FW) also did not exhibit any significant differences among the treatments (*p* = 0.469); similarly, there were no notable variations in the DMC (Dry Matter Content) of the fruits among treatments (*p* = 0.729), as shown in Table 1.

### 2.3. Impact of Fermented Kiwifruit Biomass on Tomato Fruit Quality Attributes

The results of statistical analysis on tomato fruit skin and juice color are reported in Table 2.

The statistical analysis evidenced that the FKB treatment significantly influenced the tomato skin lightness (L*) (*p* = 0.010), with the Control group exhibiting the highest L* values (35.79 ± 0.46), while a reduction of approximately 5% was observed in fruits from plants treated with 50FKB (34.20 ± 0.27). Conversely, fruits from FKB-treated plants demonstrated a more intense red color (significantly higher a* values), showing an average increase of 11% compared to the Control (21.62 ± 0.40 vs. 19.42 ± 0.55, respectively). Additionally, the b* parameter resulted significantly higher (*p* = 0.005) for fruits from the treatment 100FKB (+12%); whereas, the analysis of juice color parameters did not reveal any statistically significant differences among treatments (Table 2).

When the fruit firmness was evaluated, statistically significant differences among treatments were observed (*p* = 0.028). Specifically, fruits from plants treated with 100FKB showed higher firmness compared to those from the other treatments (Table 3).

The statistical analysis revealed significant differences in Total Soluble Solid (TSS) content (*p* = 0.008), with fruits harvested from Control plants having higher °Brix values than those harvested from plants treated with 100 mL L^−1^ of FKB (Table 3).

Regarding titratable acidity (TA), fruits harvested from 100FKB-treated plants showed lower acidity than those from the Control and 50FKB treatments (*p* = 0.016), as reported in Table 3.

The TSS/TA ratio was not influenced by the applied treatments, ranging from a minimum of 12.28 ± 0.93 to a maximum of 15.11 ± 0.58, without showing statistically significant differences among treatments (*p* = 0.067).

In terms of electrical conductivity (EC), statistical analysis indicated significant differences (*p* = 0.002), with fruits from the Control group having higher values compared to those treated with FKB in accordance with the TSS results (Table 3).

The pH of the fruit juice did not exhibit a statistically significant difference between the Control and treated groups (*p* = 0.077), showing values between 4.55 and 4.65.

Regarding the total phenolic content (TPC) among the three different treatments (Control, 50FKB, and 100FKB), the statistical analysis showed no statistically significant differences (*p* = 0.378), while interesting differences were detected in the antioxidant activity (*p* = 0.022). Specifically, statistical analysis revealed significantly higher antioxidant activity in fruits from plants treated with FKB (50FKB and 100FKB) compared to the Control group, with respective values of 3.11 ± 0.13 mM TEAC and 2.91 ± 0.23 mM TEAC versus 2.34 ± 0.16 mM TEAC, with an increase on average of 28% (Table 3).

The statistical analysis of lycopene content (LC) in the fruits demonstrated a significant enhancement, with a 57% increase observed in fruits treated with FKB, independently of the applied concentration (Table 3).

**Figure 2:**
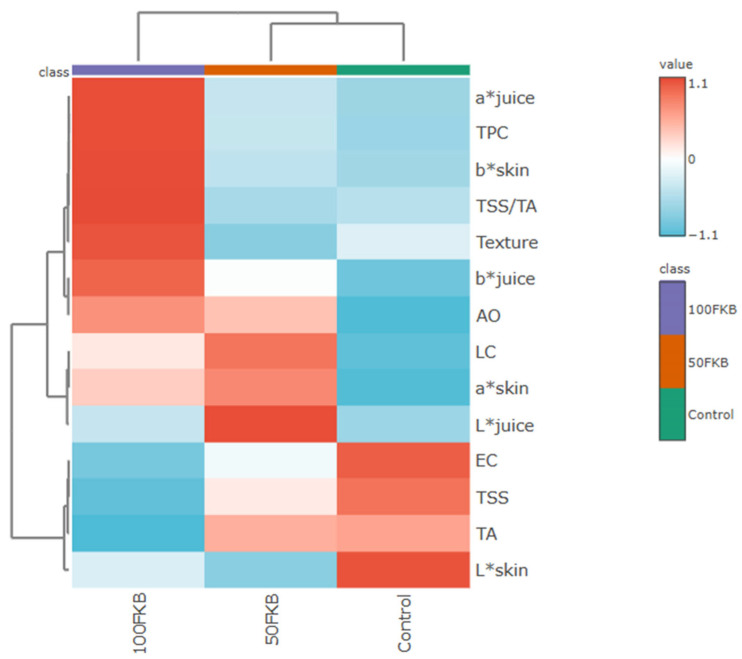
Heatmap based on Pearson’s distance showing the relationship between fruit quality parameters and different concentrations of a fermented kiwifruit biomass (Control: 0 mL L^−1^ fermented kiwifruit biomass (FKB); 50FKB: 50 mL L^−1^ of FKB; 100FKB: 100 mL L^−1^ of FKB) applied every two weeks to soilless-grown tomato plants (cv. Solarino). Different color intensities indicate the degree of correlation, with red representing positive correlations and blue representing negative correlations.

The hierarchical clustering heatmap (Figure 2), based on Pearson’s distance, revealed a clear separation among the three treatments (Control, 50FKB, 100FKB). The 100FKB group clustered independently and was characterized by higher values for several quality parameters, including a* juice, TPC, b*skin, TSS/TA, Texture, b*juice, AO, and a* skin. The 50FKB treatment occupied an intermediate position, displaying partially overlapping features with both Control and 100FKB. In particular, this concentration of the experimental biostimulant was associated with higher values of LC, a* skin, and L* juice. The Control samples formed a distinct cluster, showing relatively higher correlations with TA, TSS, and L* skin compared to the other treatments.

### 2.4. Composition of Soil Microbiome

DNA extracted from soil samples was subjected to amplicon sequencing of the v3-v4 region of 16S rDNA. After processing and quality filtering, 361,515 raw reads were obtained, with a median of 83,809 reads per sample.

**Figure 3:**
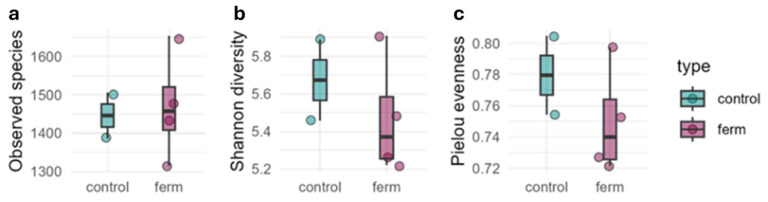
Alpha diversity of soil microbiota in Control (*n* = 2) and fermented-kiwifruit-biomass-treated (FKB) (*n* = 4) samples. Panels: (**a**) Observed species, (**b**) Shannon index, (**c**) Pielou’s evenness. Boxes represent interquartile range, center lines indicate medians, whiskers extend to 1.5× IQR, and points denote individual samples. Abbreviations: Control: 0 mL L^−1^ fermented kiwifruit biomass (FKB); ferm: 50 mL L^−1^ of FKB and 100 mL L^−1^ of FKB.

Alpha diversity metrics, including Observed species richness, Shannon diversity, and Pielou’s evenness (Figure 3), were statistically indistinguishable between Control and fermented-biostimulant-treated substrate (Kolmogorov–Smirnov test, *p* > 0.05). This suggests that the application of the fermented biostimulant did not alter within-sample richness or evenness under the conditions tested.

**Figure 4:**
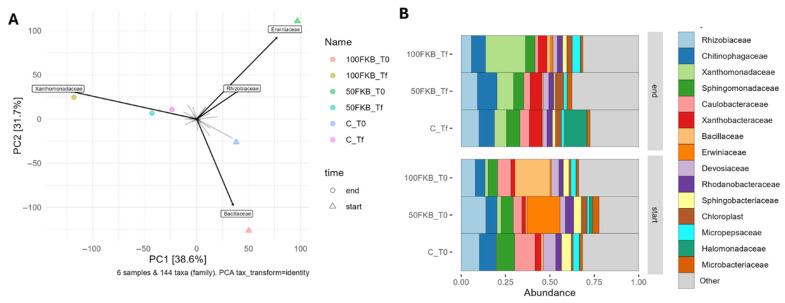
(**A**) Beta-diversity ordination of soil bacterial communities at the family level using PCA. The top four families (by abundance) are displayed as biplot vectors. (**B**) Relative abundance of the most represented bacterial families in soil samples. Faceting is used to group treatments across timepoints (T0: start and Tf: end). Abbreviation: C: Control: 0 mL L^−1^ fermented kiwifruit biomass (FKB); 50FKB: 50 mL L^−1^ of FKB; 100FKB: 100 mL L^−1^ of FKB.

Beta diversity analysis revealed a significant temporal structuring of the soil bacterial communities, whereas the biostimulant treatment did not produce a detectable shift in overall composition. In a two-factor PERMANOVA, time proved to be a significant driver of substrate microbial community (R^2^ = 0.394, F = 2.95, *p* = 0.011), while treatment proved to be not significant (R^2^ = 0.204, F = 1.53, *p* = 0.156). Consistent with these statistics, samples separated primarily along the temporal gradient in the ordination space (Figure 4A), with only partial overlap across time points, whereas treatment groups overlapped within each time point. Biplot vectors for the top four families were aligned with the temporal axis, suggesting that turnover in a subset of dominant lineages underlies the time-associated differences.

The barplot of relative abundances at the family level (Figure 4B) revealed marked differences in community composition between the initial (T0) and final (Tf) sampling points. The initial substrate microbiota is dominated in the Control samples by *Caulobacteraceae* (11.6%), *Rhizobiaceae* (10.3%), and *Sphingomonadaceae* (10.1%); while the samples treated with the FKB showed high proportions of *Erwiniaceae* (18.6% in 50FKB_T0) and *Bacillaceae* (19.8% in 100FKB_T0).

By the end of the experiment, the community composition shifted: *Xhantomonadaceae* responded to FKB addition by increasing their relative abundance (22.5% in 100FKB_Tf, 9.3% in 50FKB_Tf, 6.8% in C_Tf), and this microbial group also drove the final sample clustering in the PCA plot (Figure 4A).

**Figure 5:**
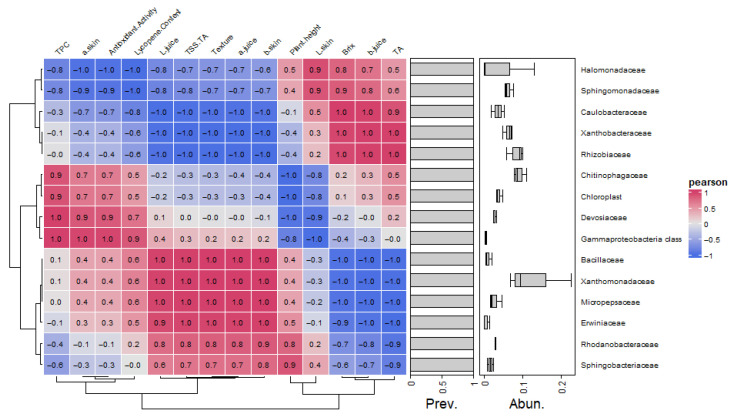
Correlation heatmap between bacterial families (top 15 at the family level) and plant/fruit traits at the final sampling time. Pearson correlation was computed. Colors denote Pearson’s r (blue = negative, red = positive; scale −1 to +1). Rows and columns are hierarchically clustered. Right panels show prevalence and relative-abundance distributions per family.

The correlation between detected microbial families and various traits was evaluated at harvest time (Figure 5). A set of families, most notably *Sphingobacteriaceae*, *Rhodanobacteraceae*, *Erwiniaceae*, *Micropepsaceae*, and *Xanthomonadaceae*, showed positive correlations (typically r ≈ 0.6–0.9) with fruit-quality traits, including total phenolics (TPC), the a* color coordinate of both skin and juice, and Texture; the same families tended to correlate negatively with titratable acidity (TA) and °Brix (often r ≈ −0.6 to −0.8). Conversely, the families of *Halomonadaceae*, *Sphingomonadaceae*, *Caulobacteraceae*, *Xanthobacteraceae*, and *Rhizobiaceae* were positively associated with °Brix, TA, and b* juice. Despite the prevalence of these genera among all samples, their spread in relative abundance and the small number of samples (*n* = 3) suggest that these associations should be viewed as exploratory. To assess whether microbial composition influences substrate microbiome functionality, correlations between the 15 most abundant bacterial families and metabolite utilization patterns determined by GenIII microplate analysis were evaluated (Figure S1). Significant correlations (*p* < 0.05) were found between the utilization of certain substrates and the composition of the rhizosphere microbiome. For instance, *Xanthomonadaceae* are positively associated with L- phenylalanine utilization, as well as with L- arginine and other amines (putrescine, phenylethylamine). Conversely, the families *Chitinophagaceae*, *Devosiaceae,* and *Sphingomonadaceae* are involved in D-malic acid utilization. Finally, it is noteworthy to mention the significant correlation between *Caulobacteraceae* and D-glucosaminic acid, an amino-sugar, and between *Halomonadaceae* and L-threonine.

## 3. Discussion

This study evaluated the effect of a biostimulant obtained from fermented kiwifruit biomass (FKB) on the growth, yield, fruit quality, and rhizosphere microbial community of soilless-grown tomato plants (cv. Solarino). The results of this study indicate that the effects of FKB were mainly expressed in fruit quality attributes rather than in yield-related parameters, which are discussed in detail below.

### 3.1. Biostimulant Properties of Fermented Kiwifruit Biomass: Experimental Considerations

Among microorganisms employed in fermentation-based valorization processes, *Lactiplantibacillus plantarum* is widely recognized for its metabolic versatility and ability to generate bioactive compounds from different plant-derived substrates [24,25]. As a member of the lactic acid bacteria (LAB) family, it efficiently converts carbohydrates into organic acids and produces a variety of secondary metabolites that can enhance the functional properties of agri-food by-products [17,26,27]. Previous studies have shown that fermentation with *L. plantarum* can substantially modify the biochemical profile of fruit- and vegetable-derived matrices, increasing their biostimulant potential [22,23].

The same fermented kiwifruit–based products were previously shown to improve morpho-physiological traits and fruit quality in hydroponically grown strawberry plants at 50 mL L^−1^ [22]. Since biostimulant effects are known to be species-specific [28], the present study tested this concentration on tomato plants and included a higher dose (100 mL L^−1^) to evaluate potential dose-dependent responses. This approach aligns with best management practices for biostimulant use, following the “four Rs” principle, “applying the Right Product, at the Right Rate, at the Right Time, and In the Right Place” [29].

Unlike foliar applications frequently reported in the literature [30,31], FKB was supplied directly to the root zone at collar level, mimicking standard fertigation practices. This choice ensured homogeneous distribution and is supported by evidence suggesting that soil or substrate application maximizes biostimulant efficacy by directly interacting with the rhizosphere environment [30,32]. Moreover, distributing the experimental biostimulant directly in the substrate, as performed in this study, ensured a uniform distribution across all plants. Indeed, because the studied variety is indeterminate and reaches considerable height by the end of the experiment, manual spray applications would not have ensured homogeneous product coverage.

In a previous study, Kisvarga et al. [33] tested an alfalfa-based biostimulant (BJ), both fermented and non-fermented through lactic fermentation, and interesting results were observed for both types of products. However, the fermented product proved to be more stable at room temperature and exhibited improved nutritional characteristics, due to the conversion of sugars, present at high levels in the non-fermented product, into organic acids. Moreover, a study conducted by Galaverni et al. [23] demonstrated that the non-fermented kiwifruit juice, used as a biostimulant in open-field conditions on the determinate tomato variety Heinz 1301, obtained simply by pressing and sterilizing the fruits, exhibited negligible biostimulant activity compared with the fermented counterparts. This could be attributed to the fermentation step, which is responsible for generating the active metabolites (e.g., organic acids, peptides, and microbial signalling molecules) that underpin the biostimulant effect. Taken together, these findings support the decision to use only the fermented kiwifruit by-product in the present study, without including a non-fermented control.

### 3.2. Effects on Vegetative Growth and Yield

FKB induced a temporary stimulation of vegetative growth during the flowering stage, particularly at the higher concentration tested. This transient effect on plant height suggests a modulation of growth dynamics rather than a sustained enhancement of vegetative development. Similar responses have been reported for other plant-based biostimulants [34], and may be partially explained by the presence of organic acids derived from lactic fermentation, which can influence nutrient uptake or cell expansion processes through pH-mediated mechanisms [35]. However, further physiological evidence would be required to confirm such mechanisms in this context.

In the present study, no significant effects were observed on stem diameter, leaf number, or total yield, although plants treated with 100FKB showed a tendency toward higher fruit number and yield compared with the Control and 50FKB treatments. This trend suggests that application frequency or timing may influence productive responses and should be further investigated in future studies. Similar outcomes have been reported in previous studies, showing that not all biostimulants influence structural growth parameters or yield, particularly under optimal cultivation conditions [23,36,37].

Yield is a key trait from a commercial perspective, and several studies report positive effects of plant-based biostimulants on fruit yield. For instance, moringa leaf extracts have been shown to enhance yield and quality in tomato and lettuce [38,39]. However, other studies reported no significant yield responses, as observed by Fusco et al. [40] on processing tomato and by Galaverni et al. [23] for fermented-kiwifruit-based biostimulants applied to open-field tomato under optimal irrigation conditions.

### 3.3. Effects on Fruits Morphology and Primary Quality Traits

FKB application did not affect fruit size, fresh weight, or dry matter content, indicating that the biostimulant did not alter fruit structural development. Similar outcomes have been reported for protein hydrolysate- and plant-extract–based biostimulants in tomato [41]. These results further support the interpretation that FKB primarily influences metabolic and biochemical traits rather than fruit morphology.

### 3.4. Effects on Fruit Color and Firmness

Fruit color represents a key commercial attribute for fresh-market tomatoes, as redness strongly influences consumer perception and purchase preference [42]. In this study, FKB-treated plants produced fruits with significantly higher a* values and reduced lightness (L*), indicating a more intense red skin color. This effect was consistent across both concentrations, although slightly more pronounced at 50FKB. These findings suggest that the application of FKB has a notable effect on the pigmentation of tomato skin, potentially indicating alterations in biochemical pathways related to color development. Moreover, as mini plum tomatoes are not only consumed fresh but also processed into products such as tomato sauce [43], the color of the juice was also evaluated. However, statistical analysis did not show significant differences among the treatments.

Fruit texture is another key attribute affecting consumer perception and postharvest performance, as firmness contributes to resistance against mechanical damage during harvesting, handling, and transportation [44,45]. Interestingly, in this study, with tomatoes harvested from plants treated with 100FKB showing higher firmness compared with Control fruits (8.02 ± 0.22 N vs. 7.25 ± 0.21 N). This response highlights a potential additional benefit of FKB on fruit quality beyond color enhancement.

### 3.5. Effects of Fermented Kiwifruit Biomass on Fruit Chemical Quality Traits

Total soluble solids (TSS), titratable acidity (TA), and their ratio were evaluated as key indicators of tomato flavor and ripeness. In the present study, a moderate decrease in TSS (approximately −6%) was observed in fruits harvested from plants treated with 100FKB compared to the Control (8.56 ± 0.09 vs. 9.12 ± 0.14 °Brix). Similar stability or slight reductions in TSS have been reported following the application of plant-derived biostimulants obtained from fennel and lemon processing residues [46], whereas other studies observed increased TSS in response to alfalfa-based biostimulants [47,48]. These contrasting results highlight that biostimulant effects on sugar accumulation strongly depend on product composition and processing methods [49].

Titratable acidity was not affected by the 50FKB treatment, while a slight decrease was observed in fruits from plants treated with 100FKB. Dasgan et al. [37] showed a general increase in TA in fruits harvested from tomato plants treated with various biostimulant formulations, highlighting that they can influence fruit acidity, although their effects can vary considerably depending on the active compound and mode of action.

Another trait related to fruit quality that did not show statistically significant differences after treatment with FKB at both concentration levels was the TSS/TA ratio. This parameter is important as it provides information on the balance between total soluble solids, mainly representing sugars and other nutrient compounds, and titratable acidity, indicating the acidity of the fruit, due to the organic acids [50]. The stability found in the TSS/TA ratio can indicate good ripeness and a proper balance between sweetness and acidity in the fruit, which is often associated with better sensory and organoleptic quality.

Fruit electrical conductivity (EC), which reflects the ability of the fruit matrix to conduct electric current and is influenced by factors such as ionic composition, soluble solids content, and tissue structure [51], was lower in fruits from FKB-treated plants compared to the Control, consistent with the pattern observed for TSS. In contrast, in a soilless tomato trial, Dasgan et al. [37] reported a general increase in fruit EC following the application of different biostimulant products. This divergence further suggests that the effect of biostimulants on EC is not universal but strongly dependent on both the type of product and the cultivar-specific physiological response.

Finally, tomato juice pH values were not affected by FKB application and remained within the range reported for commercial tomato cultivars [52].

### 3.6. Antioxidant Activity and Lycopene Accumulation

In this study, the use of FKB at two different concentrations did not significantly affect the total phenolic content of tomato fruits. Previous studies have shown that biostimulants can enhance secondary metabolism in various crops, leading to an increase in phenolic compounds; for example, potato peels and apple residual pulp improved total phenolic content in lettuce [53], garlic-based extracts increased phenolics in faba beans [54], and moringa leaves-based extracts enhanced phenolic content in coriander [55].

In the present study, fruits from both 50FKB and 100FKB plants showed an increase in antioxidant activity compared to Control fruits. This finding is noteworthy given the importance of antioxidant activity in determining both the nutritional quality and shelf- life of tomato fruits [56]. However, although several studies demonstrated that plant-derived extracts can stimulate antioxidant activity in tomato fruits [57,58], there is currently no clear evidence on how these or other botanical extracts specifically affect the antioxidant activity of fruits harvested from treated plants

The enhanced antioxidant capacity in treated fruits was directly associated with an increase in lycopene content. This biochemical response is consistent with the colorimetric changes described, as tomato fruit redness has been consistently reported to be positively correlated with lycopene concentration, with higher a* values and lower L* values reflecting increased lycopene accumulation [59,60]. Specifically, lycopene content increased by 57% in fruits from FKB-treated plants, consistent with previous findings. Lakshmi et al. [61] highlighted that biostimulant application can enhance tomato fruit quality by stimulating the metabolic pathways responsible for lycopene synthesis through bioactive compounds present in the biostimulant. Similarly, Russo et al. [62] reported a substantial increase in total carotenoids and lycopene in tomatoes treated with an experimental biostimulant derived from orange waste, while Mzibra et al. [63] observed an increase in lycopene content in greenhouse-grown tomatoes treated with a seaweed-based biostimulant, resulting in the expected enhancement of red color, as also observed in the present study. Consistent with its composition, FKB shows a relatively low content of primary nutrients (N, P, K) [22], confirming that the biostimulant effect is not due to a direct nutritional supply. Instead, the fermentation process with lactic acid bacteria is expected to generate a pool of bioactive metabolites (organic acids, peptides, exopolysaccharides, etc.) capable of modulating secondary metabolism, as reported by Bákonyi et al. [64]. This interpretation is supported by the concomitant increase in lycopene content and antioxidant activity observed in the present study.

Finally, the heatmap analysis (Figure 3) provided an integrated overview of the responses to FKB application, highlighting the dose-dependent effect, with the highest concentration tested showing the most pronounced positive impact, while the lowest concentration displayed an intermediate behavior compared to the Control.

### 3.7. Rhizosphere Microbial Responses to Fermented Kiwifruit Biomass

Regarding the influence of FKB on the microbial composition of the substrate, families such as *Rhizobiaceae* and *Chitinophagaceae* remained consistently abundant across all treatments, whereas others (e.g., *Xanthomonadaceae*, *Bacillaceae*) showed marked temporal variation. In biostimulant-treated substrates (50FKB and 100FKB), the final samples showed an increased representation of families associated with nutrient cycling (*Xanthomonadaceae*, *Caulobacteraceae*), while the Control substrate retained a higher proportion of *Rhizobiaceae.* The enrichment in *Caulobacteraceae* and *Xanthobacteraceae* in the FKB-treated substrates (50FKB and 100FKB) suggests a stimulation of microbial taxa involved in nutrient cycling, particularly nitrogen turnover. Indeed, these taxa are correlated with an increased metabolic activity toward amino acids and amines from the soil microbiome activity. Nevertheless, it is noteworthy to remember that members of the *Xanthomonadaceae* family are known as the cause of the bacterial spot defect in tomato [65]. This microbial family was present in the soil from the beginning of cultivation, and increased upon exposure to the FKB, although the defect was not encountered in the present study, and other studies report the prevalence of members of the same bacterial family in the soil microbiome of tomato [66], suggesting it might be present in the tomato-associated microbiome.

Members of *Caulobacteraceae* are known to promote biological nitrogen cycling by producing enzymes responsible for nitrogen assimilation [67], potentially enhancing nutrient availability within the rhizosphere. This functional shift may contribute to improved metabolic efficiency in plants, supporting the observed enhancement of fruit quality traits without significant changes in total yield. These patterns suggest that biostimulant application may influence the relative dominance of specific *taxa* rather than overall diversity, with potential implications for functional processes in substrate ecosystems. In line with this interpretation, a study conducted by Hadj Saadoun et al. [68] using *Lactiplantibacillus plantarum*–fermented lettuce applied to strawberry plants reported changes in soil microbial metabolic activity, as assessed by Biolog EcoPlate, with substrate utilization patterns varying in relation to treatment and plant developmental stage. In this context, it is worth noting that modern domesticated tomatoes have lost part of their microbial functional diversity compared to wild relatives [69]. Therefore, biostimulants that foster microbial enrichment in the rhizosphere, such as fermented kiwifruit biomass, may help in reestablishing functional microbiome traits associated with nutrient cycling and stress resilience, contributing to a more sustainable and balanced plant–microbe interaction.

## 4. Materials and Methods

### 4.1. Plant Material, Growing Conditions, and Experimental Setup

The experiment was conducted during the spring–summer season of 2023, from mid-May to the end of July, for a total duration of 12 weeks. The growing season in the non-climate-controlled greenhouse was characterized by typical spring–summer conditions. According to temperature data provided by the farm’s environmental sensors mean minimum and maximum air temperatures recorded during the experimental period were approximately 12–24 °C in May, 16–29 °C in June, and 19–32 °C in July. Nighttime temperatures inside the greenhouse did not fall below 16 °C throughout the trial.

The tomato cultivar used was Solarino (Rijk Zwaan-RZ, Cesena, Italy), a red mini-plum type with an average fruit weight of 8–12 g well adapted to soilless cultivation.

**Figure 6:**
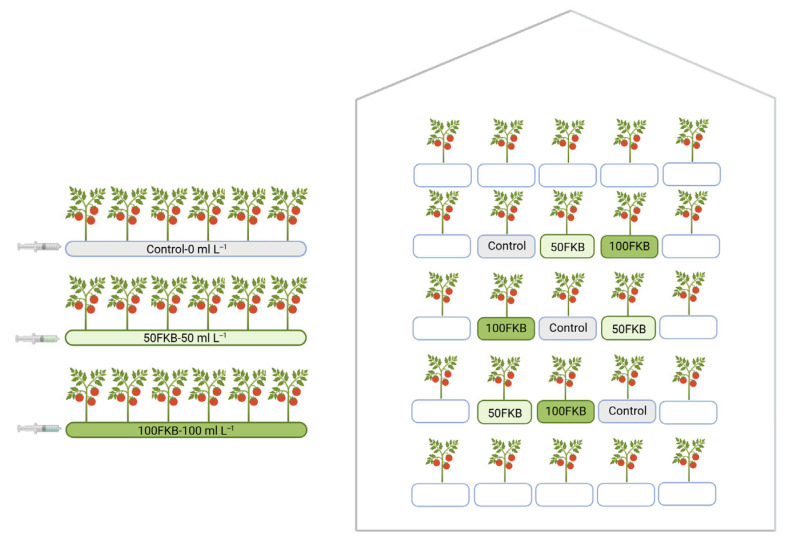
Representation of the experimental design carried out in the greenhouse, with three treatment blocks arranged on three separate central rows of the greenhouse to avoid lateral effects. The fertigation system was the same for all treatments, as shown in the scheme. Treatments consisted of 50FKB (50 mL L^−1^ fermented kiwifruit biomass), 100FKB (100 mL L^−1^ fermented kiwifruit biomass), and the Control (0 mL L^−1^ fermented kiwifruit biomass).

Seeds were sown in a local nursery in April 2023, and six-week-old seedlings at the four-leaf stage were transplanted into a greenhouse belonging to the farm Azienda Agricola Anzola Achille e Stefania (Boretto, Reggio Emilia, Italy; 44.8986, 10.5274). Plants were grown in soilless growth bags (Ageon Srl, Borgo San Dalmazzo, CN, Italy) filled with a coconut fiber substrate. Each bag (100 × 24 × 12 cm) contained a 2 kg slab of dried coconut fiber and hosted six tomato plants. The growth bags were arranged along three cultivation lines inside the greenhouse, with treatments alternated across lines to minimize positional effects and to avoid edge-related influences as much as possible (Figure 6).

The biostimulant consisted of fermented kiwifruit (*Actinidia deliciosa* ‘Hayward’) by-products, including pulp, peel, and seeds, obtained from undersized fruits discarded from local producers and farmers in Emilia-Romagna (Italy). The by-products, were ground using a laboratory blender (Knife Mill Grindomix GM 200, Retsch GmbH, Haan, Germany). The homogenized material was then sterilized at 121 °C for 20 min prior to fermentation.

Fermentation was performed using *Lactiplantibacillus plantarum* strain 4193, belonging to the University of Parma Culture Collection (UPCC). The bacterial strain was previously revitalized in de Man, Rogosa, and Sharpe (MRS) broth (Oxoid, UK) and incubated under optimal growth conditions. The sterilized kiwifruit substrate was inoculated with the active bacterial culture and fermented at 25 °C for 28 h under static conditions, reaching a final microbial concentration of approximately 8 Log CFU g^−1^. Full details of the preparation procedure and biochemical composition of the FKB, which was also used in the study by Nazeer et al. [22], are reported therein. After fermentation, the fermented kiwifruit biomass was aliquoted and stored at −20 °C until its use.

The fermented kiwifruit biomass (FKB) was diluted in distilled water at two concentrations (50 mL L^−1^ and 100 mL L^−1^) and applied directly at plant collar, starting at transplant and every two weeks thereafter, for a total of six applications (Figure 6). For each planting hole, containing two plants, 50 mL of the FKB solution was applied, corresponding to a total of 150 mL of biostimulant per growth bag.

The experimental design therefore consisted of three treatments: (i) Control without biostimulant; (ii) 50FKB treated with FKB diluted at 50 mL L^−1^; and (iii) 100FKB: treated with FKB diluted at 100 mL L^−1^.

Each treatment included three growth bags (18 plants), for a total of 54 plants.

Representative figures of the experimental setup and plant treatments are provided in the Supplementary Materials (Figure S2).

### 4.2. Fertigation and Substrate Parameters Management

Plants were fertigated daily from two hours after sunrise until two hours before sunset, with the frequency of fertigations varying throughout the season based on plant growth and environmental factors such as light and temperature. The nutrient solution used had the following composition: Ca(NO_3_)_2_ 137 g L^−1^, KNO_3_ 12.5 g L^−1^, Fe-EDDHA 1.9 g L^−1^, KH_2_PO_4_ 20 g L^−1^, MgSO_4_ 750 g L^−1^, K_2_SO_4_ 0.07 g L^−1^, MnSO_4_ 1.19 g L^−1^, ZnSO_4_ 0.5 g L^−1^, Na_2_B_4_O_7_ 0.14 g L^−1^, CuSO_4_ 0.03 g L^−1^, and Na_2_MoO_4_ 0.06 g L^−1^. The pH and electrical conductivity (EC) of the nutrient solution were adjusted to 5.5 and 4.5 mS cm^−1^, respectively. In addition, the substrate was kept within controlled ranges throughout the experiment, with pH values between 5.5 and 6.5 and EC values between 2.5 and 3.0 mS cm^−1^. The fertigation schedule, established by the farm owners, was managed through a centralized automatic system designed to prevent water and nutrient stress in the plants.

### 4.3. Vegetative and Productive Data Collection

Plants were monitored weekly, and vegetative parameters including plant height, stem diameter, and leaf number were recorded for each treatment. Plant height was measured in cm from the stem base to the apex using a measuring tape, from transplanting until topping (6 weeks after transplanting), while stem diameter was measured in until week 8. Leaf number was recorded from the day of transplanting until the start of defoliation, 7 weeks later, which was done to promote fruit ripening. Weekly height increments were calculated up to the topping stage, to assess growth dynamics over time.

Fruits were harvested at commercial maturity, numbered, weighed and measured in size. Fresh weight, expressed in g, was measured with an electronic scale (KERN^®^ EMB 1000-2, KERN, Vicenza, Italy). The weight of the freshly harvested fruits was first recorded (W1), followed by their dry weight (W2), measured after 48 h in an oven at 70 °C. The Dry Matter Content (DMC) was then calculated using the following Equation (1):

(1) $$\;DMC\;\left(\%\right)=\frac{W2}{W1}\;\times 10$$

Fruit length and width were recorded by measuring the equatorial and longitudinal diameter of fifty fruits per treatment, using a digital caliper (Moore & Wright MW110-15DFC Fractional, Sheffield, South Yorkshire, UK).

### 4.4. Characterization of Tomato Fruits

Fifty representative fruits per treatment were collected in a mid-July harvest from the central trusses (3rd to 5th) of different plants and pooled to obtain a composite sample representative of each treatment. For colorimetric, texture, and TSS analyses, measurements were performed directly on these 50 fruits per treatment. After measuring the skin color, the same 50 fruits per treatment were squeezed to obtain juice, from which the seeds were removed using a sieve. The resulting juice was used for the determination of juice color, TA, EC, and pH. For the chemical analyses of tomato fruits, including TPC and antioxidant activity (2,2-diphenyl-1-picrylhydrazyl, DPPH assay), and LC, an additional batch of 50 fruits per treatment was collected from all plants. The fruits were homogenized using a home blender (Pimmy 200 W, Ariete, Florence, Italy) to obtain tomato pulp, which was used for the subsequent extraction and analysis procedures. More detailed descriptions of the analyses conducted are provided in the following sections.

#### 4.4.1. Colorimetric Analysis

The colorimetric properties of tomato skin and juice were assessed using a portable colorimeter (CM 2600 d, Minolta Co., Osaka, Japan). The CIE L*, a*, and b* colorimetric parameters were measured, where L* indicates lightness, a* represents redness or blueness, and b* corresponds to yellowness or greenness. For skin color analysis, measurements were taken directly on the fruit surface with the colorimeter, maintaining consistent positioning and controlled ambient lighting for accuracy. Following the skin analysis, the fruits were crushed to extract juice. A 15 mL juice sample was placed on a transparent glass plate, and CIE values were recorded at room temperature. Each measurement was performed in triplicate to ensure precision, and the average values for each treatment were calculated for further analysis.

#### 4.4.2. Texture Profile

Fruit texture was evaluated by a penetration test using a TA.XT2i Texture Analyzer (Stable Micro Systems, Godalming, UK), equipped with a 30 kg load cell and a P/3 stainless steel flat-end cylindrical probe. Tests were conducted at 1 mm s^−1^ to penetration dephts of 10 mm (pericarp) and 20 mm (pericarp + endocarp), at ambient temperature (25 ± 1 °C). Firmness was expressed as the maximum force in Newtons (N) on the fruit’s equatorial region.

#### 4.4.3. Total Soluble Solid Content (TSS)

The TSS content was measured by squeezing fifty fruits per treatment and analyzing a few drops of the extracted liquid using an optical portable refractometer (Model Hanna Instruments, Padova, Italy). The results were expressed in °Brix.

#### 4.4.4. Titratable Acidity (TA)

The TA of tomato juice was determined using the titration method described by Teka et al. [70]. Approximately 5 g of prepared tomato juice was diluted with 100 mL of distilled water, and phenolphthalein was used as an indicator. The TA was calculated by titrating the diluted juice with 0.1 N NaOH. The acid content of the tomato sample was determined using the following Equation (2):

(2) $$\%citric\;acid=\frac{V\;NaOH\;\left(\mathrm{mL}\right)\;\times \;0.1\;N\;\times \;0.064}{g\;Juice\;}\;\times \;100$$

where 0.064 is the citric acid milliequivalent factor.

Subsequently, the values of TSS and TA were used to calculate the sugar:acid ratio (TSS/TA).

#### 4.4.5. Electrical Conductivity (EC)

The EC of tomato juice samples was measured using an electrical conductivity meter (Portamess^®^ 913 X Cond, Knick Elektronische Messgeräte GmbH & Co., Berlin, Germany). The electrodes were fully immersed in the tomato juice sample, and the EC was measured until constant readings were obtained. This measurement was conducted in triplicate at room temperature (25 °C).

#### 4.4.6. pH Determination

The pH of the juice was determined using a portable pH meter (Model LLG-pH meter 5, Hyde Manchester, UK), with each sample tested in three replicates.

### 4.5. Total Phenolic Content Determination

The TPC was assessed using the Folin–Ciocalteau phenol reagent [71], with some modifications. Briefly, 250 µL of extract, obtained from 1 g of tomato pulp using 20 mL of methanol:acetone (70:30 *v*/*v*), were mixed with 1 mL of Folin–Ciocalteau phenol reagent (Sigma-Aldrich, St. Louis, MO, USA) diluted to 1/10 (*v*/*v*), and 2 mL of a 20% (*w*/*v*) sodium carbonate solution. The mixture was kept in the dark for 30 min. The absorbance was measured at 760 nm using a spectrophotometer (JASCO V-530, JASCO Corporation, Easton, MD, USA). A calibration curve was constructed using gallic acid as a reference, with concentrations ranging from 10 to 100 mg/kg (5 points), to quantify the polyphenols in the samples. All extractions were performed in triplicate, and each extract was measured three consecutive times by the spectrophotometer to ensure accuracy and reading consistency. The results for total polyphenolic content were expressed as mg GAE/kg FW. This same procedure was applied to other assays used to determine the antioxidant capacity of the extracts.

### 4.6. Evaluation of Antioxidant Activity

The AO of the fruits was determined using the 2,2-diphenyl-1-picrylhydrazyl (DPPH) radical scavenging assay [72], with slight modifications. A 100 µL aliquot of sample extract or standard solution was added to 2.9 mL of a DPPH (Sigma-Aldrich, St. Louis, MO, USA) ethanolic solution (0.05 mM) and kept in the dark for 30 min. Afterward, the absorbance at 517 nm was measured using a spectrophotometer (JASCO V-530, Easton, MD, USA). To evaluate the antioxidant capacity, 6-hydroxy-2,5,7,8-tetramethylchroman-2-carboxylic acid (Trolox) (Sigma-Aldrich, St. Louis, MO, USA) was used as a reference, preparing five different standard solutions (0.1–1 mM) for the calibration curve. Additionally, a blank consisting of 100 µL of extraction solution was analyzed under the same conditions as the samples. AO was calculated based on the radical inhibition percentage (I%), as follows: I% = [(AbsB-bsS)/AbsB]×100 where AbsB was the absorbance of the blank and AbsS was the absorbance of the sample/Trolox standard solution. Results were expressed as mM TEAC (Trolox Equivalent Antioxidant Capacity). All analyses were conducted with three consecutive measurements on each sample.

### 4.7. Lycopene Content

Lycopene was extracted using the protocol by Tambunan et al. [73] with some modifications. Starting with 1 g of tomato pulp homogenized with 10 mL of a hexane: acetone (2:1:1 *v*/*v*) solution in a plastic test tube, the mixture was continuously stirred in dark conditions for 2 h. The absorbance of the supernatant (hexane layer) containing lycopene was read at 473 nm and 502 nm using a spectrophotometer (JASCO V-530, Easton, MD, USA). Absolute hexane was used as a blank. The lycopene concentration was expressed as mg/kg using the following formula [3]:

(3) $$\;c=\frac{A}{\varepsilon \;\times \;d}$$

where: ε (1280) is 1 g lycopene molar extinction coefficient in 100 mL; A is the absorbance at 473 nm and 502 nm; d is the length of quartz cuvette in cm.

### 4.8. Soil Microbial Functional Profiling

After the first treatment was performed (T0) and at the end (TF) of the experiment, tomato plant substrates (~5 g) were collected, ~10 cm away from the stem, to first analyze the functional metabolic profile using Biolog Ecoplates™, followed by DNA extraction to understand changes in microbial population dynamics. Biolog Ecoplates are 96-well plates containing 31 different carbon substrates that permit to assess functional diversity of soil microbial communities [74]. Five grams of samples were added to 45 mL Ringer solution (VWR, Lutterworth, UK), and shaken at room temperature (22 °C) for 30 min at 200 rpm. After 20 min of settling, 1 mL supernatant was diluted in 9 mL of Ringer. A total of 100 μL of solution per well were inoculated into EcoPlates in triplicates and incubated at 30 °C. The plates were analyzed by the Microplate Reader (dual-wavelength data: OD590). Twenty-four hour was the shortest incubation time in which the highest variation was found. The analysis of data was performed using (Average Well Color Development (AWCD) as a parameter that enables to capture of an integral fingerprinting of carbon sources used. The value of AWCD was calculated according to Equation (4)

(4) $$\;AWCD=\frac{\left(C-R\right)}{n}$$

where C is the OD value of each well with a carbon source, R is the OD value of the control well (water), *n* is the number of wells with carbon sources, and the value of *n* is 31. We also evaluated the Shannon index (H) resulted from H =−ΣPi ln(Pi), where Pi = ODi/ΣODi, which is the proportional color development of the well over total color development of all wells of a plate. The number of substrates oxidized (substrate richness, SR) was calculated as the sum of the number of cells where ODi value reached 0.15 after 24 h.

### 4.9. Extraction of DNA and Metataxonomic Analysis

Total genomic DNA was isolated using the Maxwell RSC PureFood GMO and Authentication Kit in combination with the Maxwell^®^ RSC (Promega Corporation, Madison, WI, USA), with minor adjustments to accommodate soil matrices, as described in Galaverni et al. [75]. The starting material consisted of the diluted soil suspension previously prepared for Biolog analysis. Briefly, an aliquot of 2.5 mL was centrifuged at 10,000 rpm for 10 min at 4 °C, after which the supernatant was discarded. The resulting pellet was resuspended in 1 mL of CTAB buffer and transferred to a bead-beating tube (Lysing Matrix E, MP Biomedicals, Santa Ana, CA, USA). DNA extraction then proceeded according to the manufacturer’s instructions. The concentration and purity of the recovered nucleic acids were assessed using a NanoDrop spectrophotometer (NanoDrop™ 2000, Thermo Fisher Scientific, Waltham, MA, USA).

Bacterial community composition was analyzed at two time points: after transplant (mid-May) and after harvesting (end July). The V3–V4 hypervariable region of the 16S rRNA gene was amplified using standard primers and PCR conditions [76], and sequencing libraries were prepared and processed by Novogene Co., Ltd. (Cambridge, UK) on an Illumina NovaSeq 6000 platform, generating 250 bp paired-end reads.

Raw sequence quality was evaluated with FastQC [77] (https://www.bioinformatics.babraham.ac.uk/projects/fastqc/ (accessed on 25 September 2025).), and low-quality reads were filtered using Prinseq [78], removing sequences shorter than 400 bp and trimming bases below a Phred score of 20. Paired-end reads were merged with FLASH [79], and downstream analyses were performed in QIIME 2 (v. 2022.11) [80]. Amplicon sequence variants (ASVs) were delineated at 99% similarity, and taxonomic assignment was carried out using a Naïve Bayes classifier trained on the SILVA database (v. 138) [81,82,83].

Spearman correlations between CLR-transformed bacterial family abundances and AWCD-normalized GENIII substrate utilization profiles were computed and visualized as a clustered heatmap following the approach described by Galaverni et al. [75].

### 4.10. Statistical Data Analysis

One-way ANOVA was performed using General Linear Model (GLM) procedures in IBM SPSS Statistics 29.0.1.0 (SPSS Inc., Chicago, IL, USA) to analyze the following parameters: plant height, number of leaves, stem diameter, color, texture, total soluble solids (TSS), titratable acidity (TA), electrical conductivity (EC), pH, total phenolic content (TPC), antioxidant activity (DPPH assay), and lycopene content.

For growth-related variables (plant height, number of leaves, stem diameter), a repeated-measures analysis was applied to account for temporal measurements taken on the same plants. For all other parameters, one-way ANOVA was performed, and mean separation was conducted using Tukey’s test (*p* ≤ 0.05).

Plant responses to the application of FKB at different concentrations were analyzed using a heatmap generated with the MetaboAnalyst 6.0 web platform. Prior to analysis, the data were log10-transformed since the variables were expressed in different units and displayed considerable variation in scale. Pearson’s distance was calculated to assess the relation between treatments and variables based on similarity patterns. The resulting heatmap was visualized using a color gradient, with red indicating strong positive relation and blue indicating strong negative relation.

The BIOM file resulting from QIIME2 as well as the phylogenetic tree were imported in R (version 4.5.0), imported using package Phyloseq (v. 1.42.0) and subsequent analyses were performed using packages Microbiome (v. 1.30.0), and MicroViz (v. 0.12.7). The MicroViz package was used for data visualization. Amplicon sequence data were aggregated at the family level without transformation, and ordination was performed using the PCA method. Correlations between bacterial taxa and sample metadata were assessed in R using the microViz workflow, and restricting the dataset to the final time point. Taxonomic counts were aggregated at the family level, and the top 15 bacterial families were selected using the maximum abundance criterion across samples. Pairwise correlations were then computed between the abundances of the selected families and the metadata variables, and the results were visualized as a heatmap. Phenotypic raw data from Biolog analysis were elaborated with Rstudio for statistical analysis and data visualization of assays. To reduce the noise levels, all absorbance values of carbon source utilization were referred against the negative control well (A1) and subsequently, all were divided by the respective AWCD. Negative values were set to 0. Normalized data were used for statistical analysis. Spearman correlations between CLR-transformed abundances of the 15 most abundant bacterial families and substrate utilization profiles (GenIII microplate data) were computed, and significant associations (*p* < 0.05) were visualized as a clustered heatmap.

## 5. Conclusions

The results of this study highlight the importance of exploring locally available input sources to optimize resource use and reduce waste generation through the valorization of food by-products as plant biostimulants. Although the application of fermented kiwifruit biomass (FKB) did not result in a significant increase in tomato plant yield, clear and consistent improvements in fruit quality were observed. In particular, fruits from plant treated with 100FKB exhibited increased firmness, while the 50FKB and 100FKB treatments both significantly enhanced antioxidant levels. Moreover, lycopene content increased by 57% in treated fruits compared to the Control, independently of the applied concentration, and fruits from 100FKB-treated plants showed a more intense red skin coloration, indicating an improvement in visual and commercial quality. Beyond fruit quality, FKB application was associated with targeted changes in the substrate microbial composition, without affecting overall bacterial diversity. These results indicate a selective modulation of the rhizosphere microbiome, potentially supporting nutrient-related functions linked to fruit quality improvements.

These findings demonstrate that the fermentation of fruit biomass by lactic acid bacteria, in this case kiwifruit not suitable for human consumption, opens an avenue of opportunities for a sustainable circular economy. A key novelty of this study lies in the direct use of fermented biomass without additional extraction or purification steps, suggesting a simplified and potentially more sustainable approach for the exploitation of food waste in agriculture. The observed increase in tomato fruit firmness is particularly relevant from a postharvest perspective, as it may contribute to extended shelf life and reduced losses along the supply chain.

Future research should aim to further investigate the effectiveness of this experimental biostimulant under different agronomic conditions and on other crop species, as well as to optimize application rates and timing throughout specific plant developmental stages. Additionally, evaluating the effects of FKB in relation to truss position and under abiotic stress conditions could provide further insight into its mode of action and practical applicability. Overall, fermented kiwifruit biomass emerges as a promising, low-input biostimulant capable of enhancing fruit quality while supporting more sustainable and resource-efficient agricultural systems.

## Figures and Tables

**Table 1:** Influence of the fermented kiwifruit biomass on productive and morphological parameters at the end of plant cycle.

Treatment	Fruit/Plant	Yield/Plant	LD	ED	Fruit FW	DMC
	(g)	(cm)	(cm)	(cm)	(g)	(%)
Control	80.2 ± 10.6 ^a^	1095.4 ± 145.0 ^a^	3.79 ± 0.11 ^a^	2.37 ± 0.04 ^a^	13.66 ± 0.48 ^a^	7.77 ± 0.23 ^a^
50FKB	72.7 ± 5.3 ^a^	995.4 ± 72.7 ^a^	3.79 ± 0.05 ^a^	2.44 ± 0.04 ^a^	13.69 ± 0.45 ^a^	7.94 ± 0.38 ^a^
100FKB	96.5 ± 8.1 ^a^	1385.0 ± 117.2 ^a^	3.90 ± 0.05 ^a^	2.40 ± 0.05 ^a^	14.35 ± 0.37 ^a^	8.08 ± 0.14 ^a^

One-way ANOVA followed by Tukey’s test. Means followed by the same letter are not significantly different (*p* > 0.05). Abbreviations—Control: 0 mL L^−1^ fermented kiwifruit biomass (FKB); 50FKB: 50 mL L^−1^ of FKB; 100FKB: 100 mL L^−1^ of FKB; LD: Longitudinal diameter; ED: Equatorial diameter; Fruit FW: Fruit Fresh Weight; DMC: Dry Matter Content.

**Table 2:** Influence of fermented kiwifruit biomass on the tomato fruit and juice color.

Treatment	L* Skin	a* Skin	b* Skin	L* Juice	a* Juice	b* Juice
Control	35.79 ± 0.46 ^a^	19.43 ± 0.55 ^b^	18.66 ± 0.76 ^b^	34.36 ± 0.24 ^a^	8.13 ± 0.42 ^a^	9.11 ± 0.25 ^a^
50FKB	34.20 ± 0.27 ^b^	21.96 ± 0.34 ^a^	19.09 ± 0.32 ^b^	34.42 ± 0.27 ^a^	8.47 ± 0.72 ^a^	8.98 ± 0.21 ^a^
100FKB	34.83 ± 0.29 ^ab^	21.29 ± 0.46 ^a^	21.16 ± 0.47 ^a^	34.53 ± 0.38 ^a^	9.7 ± 0.30 ^a^	8.66 ± 0.24 ^a^

One-way ANOVA, Tukey’s test, *p* ≤ 0.05. Per each parameter, different letters indicate values statistically different. Abbreviation—Control: 0 mL L^−1^ fermented kiwifruit biomass (FKB); 50FKB: 50 mL L^−1^ of FKB; 100FKB: 100 mL L^−1^ of FKB.

**Table 3:** Influence of fermented kiwifruit biomass on the tomato fruit quality and biochemical parameters.

Treatment	Firmness	TSS	TA	EC	TPC	AO	LC
Control	7.25 ± 0.21 ^b^	9.12 ± 0.14 ^a^	0.66 ± 0.05 ^a^	0.74 ± 0.03 ^a^	725.43 ± 28.65 ^a^	2.34 ± 0.16 ^b^	7.56 ± 0.66 ^b^
50FKB	7.43 ± 0.21 ^b^	8.89 ± 0.10 ^ab^	0.66 ± 0.02 ^a^	0.67 ± 0.01 ^b^	841.03 ± 49.63 ^a^	3.11 ± 0.13 ^a^	11.90 ± 1.37 ^a^
100FKB	8.02 ± 0.22 ^a^	8.56 ± 0.09 ^b^	0.51 ± 0.03 ^b^	0.63 ± 0.01 ^b^	772.77 ± 23.77 ^a^	2.91 ± 0.23 ^a^	11.89 ± 1.02 ^a^

One-way ANOVA, Tukey’s test, *p* ≤ 0.05. Per each parameter, different letters indicate values statistically different. Unit of measure: Firmness expressed in N = Newton. Abbreviations—Control: 0 mL L^−1^ fermented kiwifruit biomass (FKB); 50FKB: 50 mL L^−1^ of FKB; 100FKB: 100 mL L^−1^ of FKB; Firmness expressed in Newton (N); TSS: Total Soluble Solids expressed in °Brix; TA: Titratable Acidity expressed in % citric acid; EC: Electrical conductivity expressed in mS; TPC: Total phenolic content expressed in mg/Kg; AO: Antioxidant Activity expressed in mM TEAC; LC: Lycopene Content expressed in mg/Kg.

## Data Availability

The raw data supporting the conclusions of this article are not included but will be made available by the authors upon request. The amplicon sequences were deposited into the sequence read archive (SRA) from NCBI and are available under the accession number PRJNA1372462 (https://www.ncbi.nlm.nih.gov/bioproject/1372462 (accessed on 30 September 2025)).

## Supplementary Materials

The following supporting information can be downloaded at: https://www.mdpi.com/article/10.3390/plants15010082/s1, Figure S1: Spearman correlation heatmap between the 15 most abundant bacterial families (CLR-transformed abundances) and metabolite intensities measured in GENIII assays. Figure S2: Images representative of the experimental setup used in the soilless tomato cultivation trial. Table S1: Influence of fermented kiwifruit biomass on tomato plant height recorded until topping. Table S2: Influence of fermented kiwifruit biomass on tomato plant number of leaves recorded until defoliation.
