# Supplementary material for: Fermented Kiwifruit By-Product as Experimental Biostimulant for Soilless Mini-Plum Tomato Cultivation
Source: Plants (Basel). 2025 Dec 26;15(1):82. doi: 10.3390/plants15010082 (PMC12787496; doi:10.3390/plants15010082)
Supplement: Supplementary file 1 [file plants-15-00082-s001.zip › plants-4043394-supplementary.pdf]

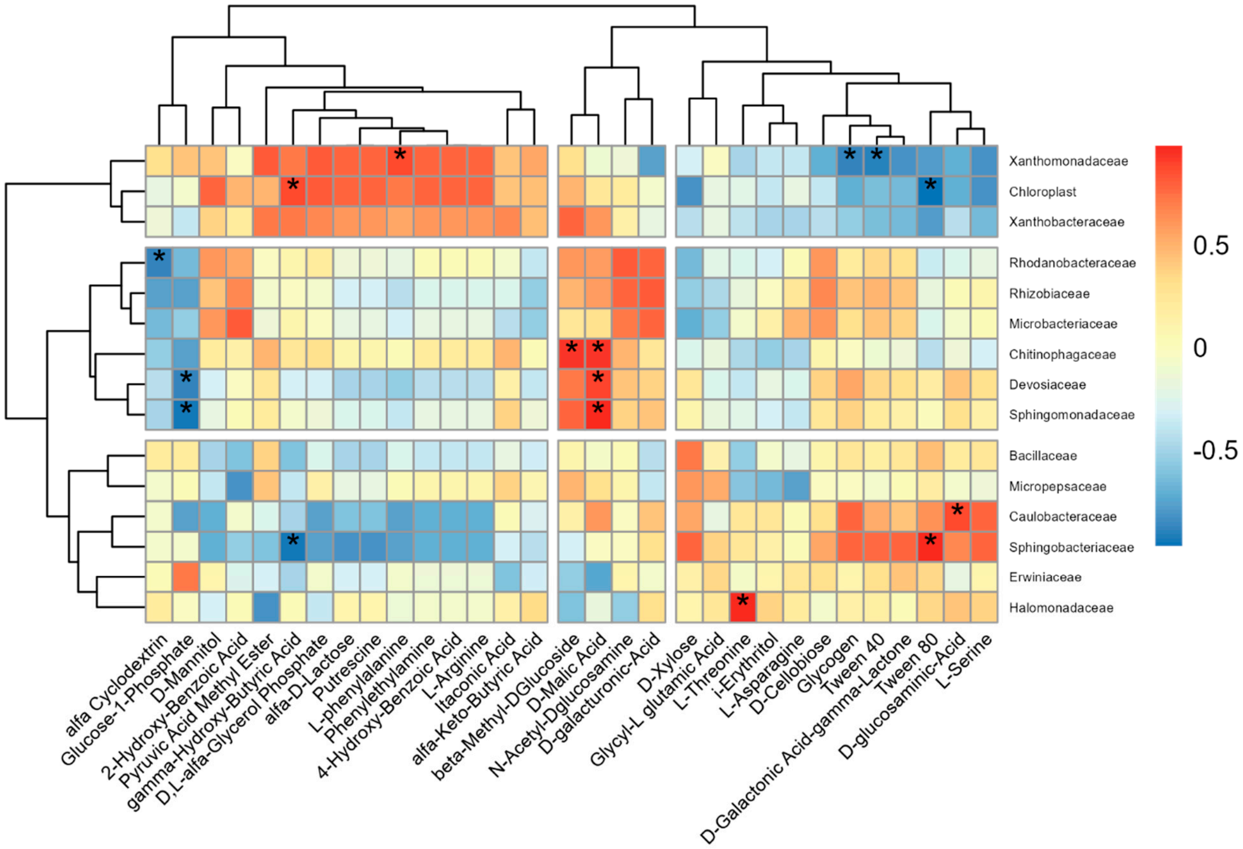

Figure S1. Spearman correlation heatmap between the 15 most abundant bacterial families (CLR-transformed abundances) and metabolite intensities measured in GENIII assays. Colors represent correlation intensity and direction (red = positive, blue = negative). Asterisks (*) indicate statistically significant correlations ($p < 0.05$). Rows correspond to bacterial families, columns to metabolites, with hierarchical clustering applied to both dimensions to highlight association patterns.

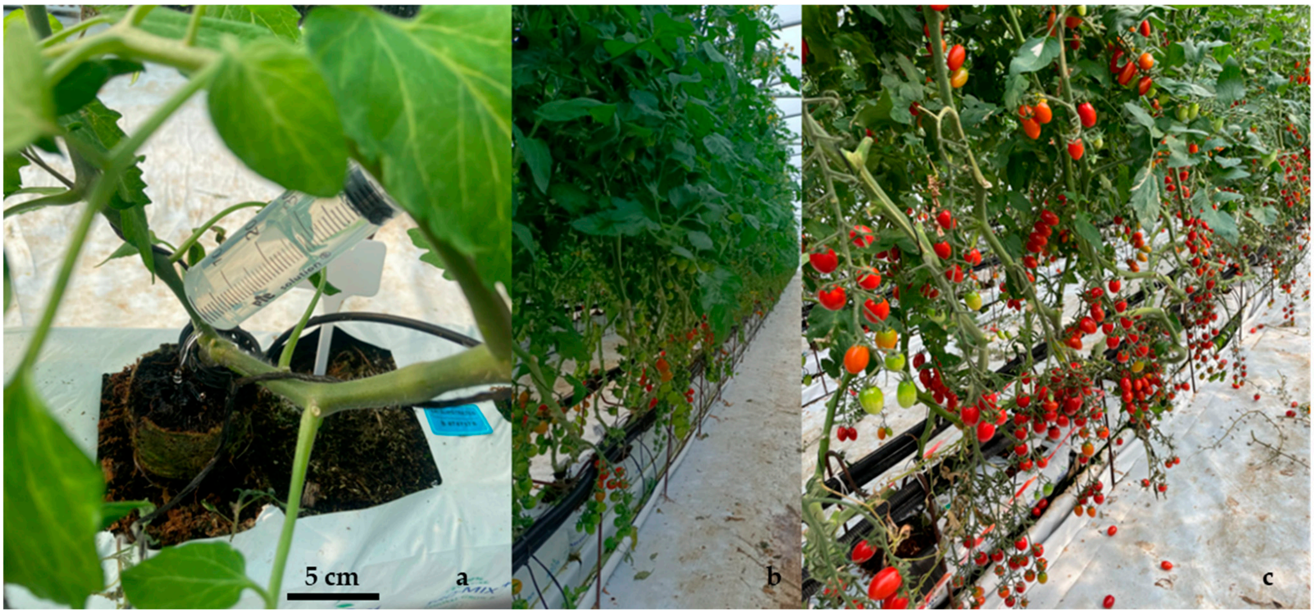

Figure S2. Images representative of the experimental setup used in the soilless tomato cultivation trial. (a) Application of the fermented kiwifruit biostimulant (FKB) using a syringe to deliver the solution directly to the growth bag substrate; (b) tomato plants at the intermediate growth stage inside the greenhouse; (c) fruit ripening stage showing clusters of plum-type tomatoes cultivated under the different treatments.

Tables

Table S1: Influence of fermented kiwifruit biomass on plant height, during the first eight weeks of tomato plant cultivation

Treatment	Week1	Week2	Week3	Week4	Week5	Week6	Week7	Week8
	(cm)							
Control	12.2±0.7 [a]	32.0±0.6 [b]	49.6±1.3 [b]	103.3±2.6 [a]	153.8±3.4 [a]	153.8±3.6 [a]	199.5±3.9 [a]	229.0±4.23 [a]
50FKB	14.0±0.3 [a]	34.0±0.7 [ab]	54.3±0.8 [a]	92.6±2.6 [b]	118.1±3.9 [a]	148.0±2.2 [a]	197.3±3.3 [a]	222.3±5.6 [a]
100FKB	12.6±0.4 [a]	35.3±0.9 [a]	53.0±1.0 [a]	99.8±0.7 [a]	122.3±2.2 [a]	158.5±5.0 [a]	184.3±13.4 [a]	229.3±4.3 [a]

One-way ANOVA, Tukey's test, $p \leq 0.05$. Within each week, different letters indicate values statistically different.

Abbreviations: Control: 0 mL L^{-1} fermented kiwifruit biomass (FKB); 50FKB: 50 mL L^{-1} of FKB; 100FKB: 100 mL L^{-1} of FKB

Table S2: Influence of fermented kiwifruit biomass on leaves number during the first six weeks of tomato plant cultivation

Treatment	Week1	Week2	Week3	Week4	Week5	Week6
	n°					
Control	6.67±0.21 [a]	8.00±0.00 [a]	12.84±0.40 [a]	12.50±0.61 [a]	21.66±1.02 [a]	22.83±1.10 [a]
50FKB	7.00±0.00 [a]	8.34±0.61 [a]	12.50±1.02 [a]	20.84±0.94 [a]	22.66±0.61 [a]	25.00±0.73 [a]
100FKB	7.00±0.00 [a]	8.00±0.00 [a]	13.84±1.30 [a]	21.16±0.30 [a]	23.66±0.76 [a]	25.33±0.76 [a]

One-way ANOVA, Tukey's test, $p \leq 0.05$. Within each week, different letters indicate values statistically different.

Abbreviations: Control: 0 mL L^{-1} fermented kiwifruit biomass (FKB); 50FKB: 50 mL L^{-1} of FKB; 100FKB: 100 mL L^{-1} of FKB
